# Matrix Metalloproteinase Response of Dendritic Cell, Gingival Epithelial Keratinocyte, and T-Cell Transwell Co-Cultures Treated with *Porphyromonas gingivalis* Hemagglutinin-B

**DOI:** 10.3390/ijms19123923

**Published:** 2018-12-07

**Authors:** Amber M. Bates, Carol L. Fischer, Vrushali P. Abhyankar, Georgia K. Johnson, Janet M. Guthmiller, Ann Progulske-Fox, Kim A. Brogden

**Affiliations:** 1 Iowa Institute for Oral Health Research, College of Dentistry, The University of Iowa, Iowa City, IA 52242, USA; kim-brogden@uiowa.edu; 2 Department of Biology, Waldorf University, Forest City, IA 50436, USA; carol.fischer@waldorf.edu; 3 Department of Periodontology, College of Dentistry, University of Tennessee Health Science Center, Memphis, TN 38103, USA; vabhyank@uthsc.edu; 4 Department of Periodontics, College of Dentistry, The University of Iowa, Iowa City, IA 52242, USA; georgia-johnson@uiowa.edu; 5 College of Dentistry, University of Nebraska Medical Center, Lincoln, NE 68583, USA; janet.guthmiller@unmc.edu; 6 Center for Molecular Microbiology and Department of Oral Biology, University of Florida, Gainesville, FL 32603, USA; APFOX@dental.ufl.edu

**Keywords:** hemagglutinin-B, transwell co-cultures, matrix metalloproteinases

## Abstract

Matrix metalloproteinases (MMPs) are enzymes involved in periodontal tissue destruction. Hemagglutinin B (HagB) from the periodontal pathogen *Porphyromonas gingivalis* induces an elevated MMP response in dendritic cells, but responses from cultures of single-cell types do not reflect the local tissue environment. The objective of this study was to measure HagB-induced MMP responses in a transwell co-culture system containing dendritic cells, gingival epithelial (GE) keratinocytes, and CD4+ T-cells. Transwell co-cultures were assembled and treated with or without HagB. Immunoassays were used to determine production of MMP1, MMP7, MMP9, and MMP12 in response to HagB up to 64 h. Control responses were subtracted from HagB-induced responses. A two-way fixed effect ANOVA was fit to log-transformed concentrations and pairwise group comparisons were conducted (*p* < 0.05). At 64 h, dendritic cells produced elevated MMP1 and MMP9 responses, which were attenuated in the 3-cell co-culture (*p* < 0.05). There were also significant differences in MMP7 and MMP12 production between single-cell cultures and co-cultures. These results support the need to use multiple cell types in culture models to evaluate a more representative response to proinflammatory agonists. This three-cell transwell co-culture model may help us better understand the inflammatory process in periodontal disease and test novel therapeutic approaches.

## 1. Introduction

Periodontal disease, a chronic inflammatory disease that causes tissue damage in the oral cavity, affects approximately 46% of U.S. adults [1]. Periodontal disease results from exposure of gingival tissue to microorganisms and microbial products in adhered polymicrobial biofilms and the subsequent host inflammatory and immune responses they induce [2]. Biofilms contain a number of early and late colonizers, including *Porphyromonas gingivalis*. The prolonged inflammatory response to this biofilm results in elevated matrix metalloproteinase (MMP) production, which in turn leads to degradation of collagen fibers and subsequent periodontal attachment and bone loss, and ultimately tooth loss if left untreated. 

The periodontal inflammatory response is a complex process involving communication between cells and strict regulation of cell activities necessary for host defense [3]. A network of cytokines and chemokines directs cell migration, survival, and immune cell function. MMPs are enzymes that play a critical role in the inflammatory response either by modifying proteins post-translationally to promote rapid delivery to other cells or by inactivating these proteins to initiate or terminate the immune process [4]. These functions allow MMPs to direct systematic inflammation and regulate cytokine biosynthesis through activation of signal transduction pathways. In periodontal disease, there is an elevated MMP response associated with the inflammatory response.

Matrix metalloproteinases are major enzymes implicated in extracellular matrix degradation and tissue remodeling [5]. MMPs are part of a large family of calcium-dependent, zinc-containing endopeptidases categorized into six groups based on their respective substrates [6,7]. Healthy adults express low levels of MMPs compared to the elevated levels expressed in adults with inflammatory diseases [6]. MMPs play an important role in the physiological response during wound healing, tissue repair, morphogenesis, tooth eruption, cell communication, and remodeling after injury, and they are also implicated in diseases such as rheumatoid arthritis, atherosclerosis, cancer, and periodontal disease. With respect to periodontal disease, biofilm induced inflammation amplifies MMP expression leading to increased tissue damage.

Recently, we reported that dendritic cells produce an enhanced cytokine and MMP response when treated with Hemagglutinin B (HagB), a virulence factor of the periodontal pathogen *P. gingivalis* [8]. Because cells in single-cell culture systems cannot receive signals from surrounding cells as they would in the natural host environment, the results from single-cell culture studies are not representative of the host response. Thus, studies encompassing multiple cell types are needed to better represent the *in vivo* host response. In this study, we utilized a three-cell transwell co-culture, composed of dendritic cells, gingival epithelial (GE) keratinocytes, and CD4+ T-cells, three cell types that play an important role in the innate immune response in periodontal disease. CD4+ helper T-cells were included in the transwell co-culture because they are the most dominant type of T-cell in the gingiva and have been suggested to play roles in homeostasis and immunopathology [9,10]. Dendritic cells in the periodontal tissues detect bacteria and initiate an immune response by migrating to the lymph nodes, leading to activation of other cell types and their recruitment to these tissues [11].

The transwell co-culture allows for the exchange of extracellular biomarkers from one cell type to stimulate co-cultured cells in an allogeneic system. In this study, we explored the use of immune cells and epithelial cells from different hosts in a three-cell transwell co-culture where the cells do not have physical contact with one another. Our study compared the *P. gingivalis* HagB-induced MMP response in a uniquely stacked, three-cell transwell co-culture system containing dendritic cells, GE keratinocytes, and T-cells to the MMP response from single-cell cultures.

## 2. Results

### 2.1. Experimental Set-Up of the Three-Cell Transwell Co-Culture.

**Figure 1:**
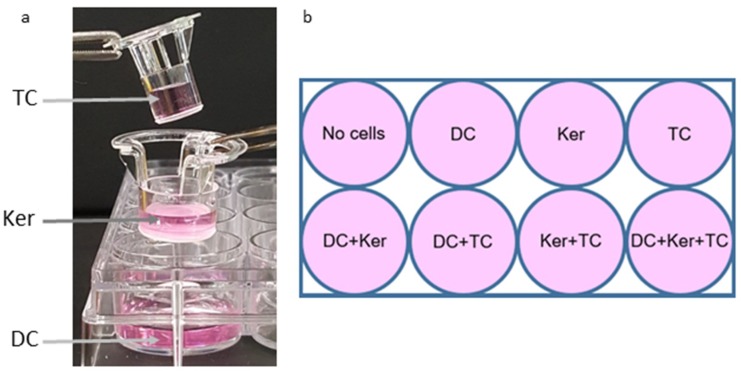
(**a**) Set-up of the three-cell transwell co-culture. The top insert contained CD4+ T-cells (TC), the middle insert contained GE keratinocytes (Ker) and the bottom well contained dendritic cells (DC). Cells were suspended in LGM-3. The inserts stack into each other. A porous membrane is found on the bottom of each insert, allowing small molecules, such as cytokines and MMPs, to travel between the cell layers while cells remain in their designated tiers. This plate was inserted in the incubator on a shaker plate to facilitate diffusion between the inserts. (**b**) Template used to establish the one-cell, two-cell, and three-cell co-cultures. Two plates were constructed; one was treated with HagB (test) and the other with diluent (control).

**Figure 2:**
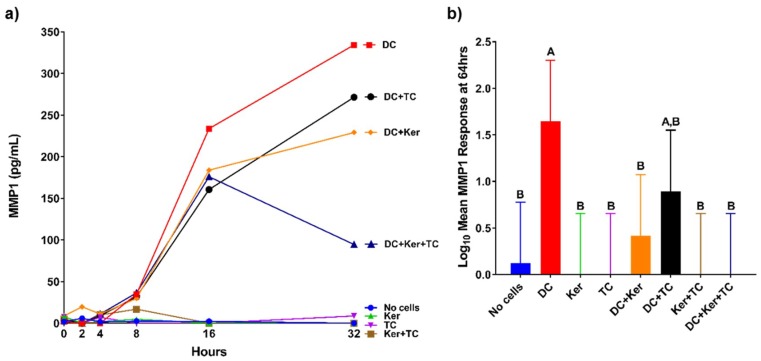
(**a**) Matrix metalloproteinase 1 (MMP1) responses of single cell and co-cultures when treated with hemagglutinin B (HagB) (*n* = 3). HagB was added at Time 0 to single-cell cultures and co-cultures of T-cells (TC), GE keratinocytes (Ker), and dendritic cells (DC). MMP1 concentrations were determined at 0, 2, 4, 8, 16, and 32 h. The MMP1 responses from the HagB diluent were subtracted from the HagB-induced MMP1 responses. (**b**) MMP1 responses to HagB at 64 h (*n* = 9). The MMP1 responses from the HagB diluent were subtracted from the HagB-induced MMP1 responses. A two-way fixed effect ANOVA was fit to log-transformed concentrations of MMP1. Pairwise group comparisons were conducted using the post-hoc Tukey’s honest significance test. A 0.05 level was used determine statistically significant differences between groups. Shared letters above bars indicate the groups are not statistically different from one another (*p* > 0.05), while differing letters indicate significantly different mean values (*p* < 0.05).

**Figure 3:**
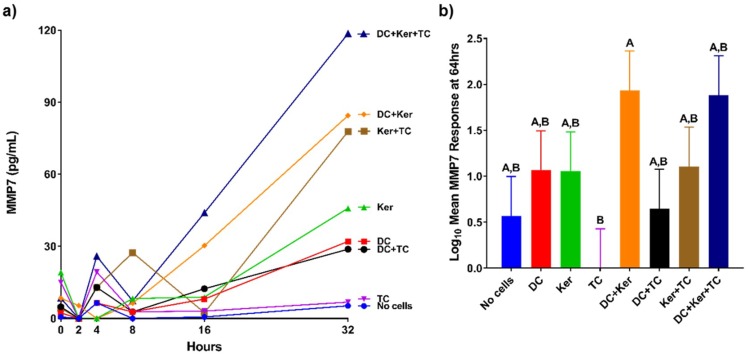
(**a**) MMP7 responses of single cell and co-cultures when treated with HagB (*n* = 3). HagB was added at Time 0 to single-cell cultures and co-cultures of T-cells (TC), GE keratinocytes (Ker), and dendritic cells (DC). MMP7 concentrations were determined at 0, 2, 4, 8, 16, and 32 h. The MMP7 responses from the HagB diluent were subtracted from the HagB-induced MMP7 responses. (**b**) MMP7 responses to HagB at 64 h (*n* = 9). The MMP7 responses from the HagB diluent were subtracted from the HagB-induced MMP7 responses. A two-way fixed effect ANOVA was fit to log-transformed concentrations of MMP7. Pairwise group comparisons were conducted using the post-hoc Tukey’s honest significance test. A 0.05 level was used determine statistically significant differences between groups. Shared letters above bars indicate the groups are not statistically different from one another (*p* > 0.05), while differing letters indicate significantly different mean values (*p* < 0.05).

**Figure 4:**
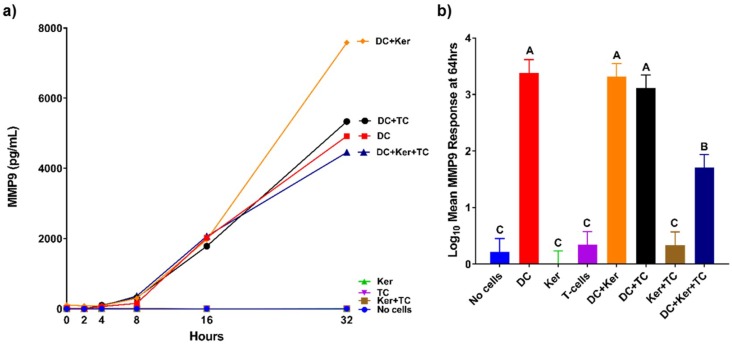
(**a**) MMP9 responses of single cell and co-cultures when treated with HagB (*n* = 3). HagB was added at Time 0 to single-cell cultures and co-cultures of T-cells (TC), GE keratinocytes (Ker), and dendritic cells (DC). MMP9 concentrations were determined at 0, 2, 4, 8, 16, and 32 h. The MMP9 responses from the HagB diluent were subtracted from the HagB-induced MMP9 responses. (**b**) MMP9 responses to HagB at 64 h (*n* = 9). The MMP9 responses from the HagB diluent were subtracted from the HagB-induced MMP9 responses. A two-way fixed effect ANOVA was fit to log-transformed concentrations of MMP9. Pairwise group comparisons were conducted using the post-hoc Tukey’s honest significance test. A 0.05 level was used determine statistically significant differences between groups. Shared letters above bars indicate the groups are not statistically different from one another (*p* > 0.05), while differing letters indicate significantly different mean values (*p* < 0.05).

**Figure 5:**
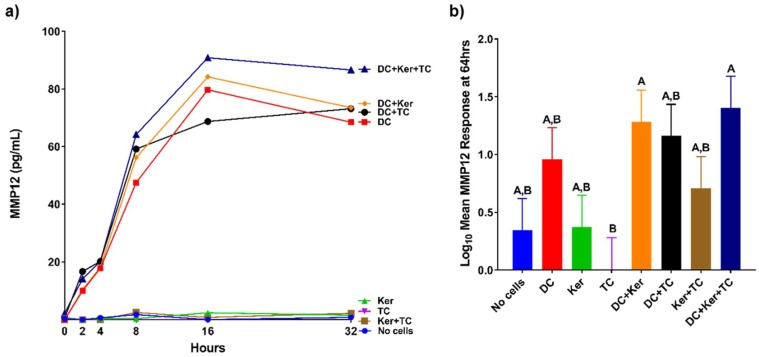
(**a**) MMP12 responses of single cell and co-cultures when treated with HagB (*n* = 3). HagB was added at Time 0 to single-cell cultures and co-cultures of T-cells (TC), GE keratinocytes (Ker), and dendritic cells (DC). MMP12 concentrations were determined at 0, 2, 4, 8, 16, and 32 h. The MMP12 responses from the HagB diluent were subtracted from the HagB-induced MMP12 responses. (**b**) MMP12 responses to HagB at 64 h (*n* = 9). The MMP12 responses from the HagB diluent were subtracted from the HagB-induced MMP12 responses. A two-way fixed effect ANOVA was fit to log-transformed concentrations of MMP12. Pairwise group comparisons were conducted using the post-hoc Tukey’s honest significance test. A 0.05 level was used determine statistically significant differences between groups. Shared letters above bars indicate the groups are not statistically different from one another (*p* > 0.05), while differing letters indicate significantly different mean values (*p* < 0.05).

The production of MMPs in response to HagB was assessed in a modified three-cell transwell co-culture (Figure 1). The production of MMPs was first examined in samples at 0, 2, 4, 8, 16, and 32 h after HagB addition or HagB diluent (*n* = 3, Figure 2a, Figure 3a, Figure 4a and Figure 5a, and Supplemental Table S1) and then more closely examined at 64 h (*n* = 9, Figure 2b, Figure 3b, Figure 4b and Figure 5b, and Supplemental Table S2). Note that Figure 2a, Figure 3a, Figure 4a and Figure 5a are from one experiment with three replicate reads at each time point (*n* = 3) from 0 to 32 h, while Figure 2b, Figure 3b, Figure 4b, and Figure 5b are based on three experiments with three replicate reads each (*n* = 9) at 64 h. These values were determined by subtracting the response to the HagB diluent from the HagB-induced response. This not only eliminates the response of any reaction to the diluent, but also eliminates the cell responses induced by the other cell types in the co-cultures and any baseline MMP production from the cells when no agonist is present. In most cases, MMP production was first observed from 8 to 16 h.

### 2.2. Single-Cell Culture Responses to HagB

The transwell co-cultures were set up with only one cell type. Each cell type was in its respective tier: Dendritic cells (bottom), GE keratinocytes (middle), and T-cells (top). By 64 h, HagB stimulated single cultures of dendritic cells, GE keratinocytes, and T-cells produced varying concentrations of MMP1, MMP7, MMP9, and MMP12 (Figure 2b, Figure 3b, Figure 4b and Figure 5b). Of the single-cell cultures at 64 h, dendritic cells produced significantly higher amounts of MMP1 and MMP9 in response to HagB (*p* < 0.05), while GE keratinocytes and T-cells produced little to no MMP1 and MMP9. This trend of MMP1 and MMP9 production was observed throughout the 64 h, starting at 8 h (Figure 2a and Figure 4a). There was little to no production of MMP7 and MMP12 in the single-cell cultures, as there was no significant difference (*p* > 0.05) from the no cell control at 64 h (Figure 3b and Figure 5b). However, from the detection of MMP12 from 0 to 32 h, dendritic cells were the highest single-cell culture to produce MMP12 in response to HagB. In general, GE keratinocytes and T-cells single-cell cultures produced little to no MMP response to HagB of the four MMPs examined.

### 2.3. Multiple Cell Types in the Three-cell Transwell Co-Culture System

As noted above, dendritic cells produced the highest amount of MMP1 in single-cell cultures, but when dendritic cells were cultured with GE keratinocytes or GE keratinocytes + T-cells, production of MMP1 was attenuated (*p* < 0.05). At 64 h, the dendritic cell MMP1 response was attenuated in dendritic cells + GE keratinocytes (*p* < 0.05) and dendritic cells + GE keratinocytes + T-cells transwell co-cultures (*p* < 0.05) (Figure 2b). 

Dendritic cells were also the main drivers of the MMP9 response to HagB, as all cultures containing dendritic cells had the greatest response (Figure 4). However, at 64 h the three-cell co-culture values were significantly less than the dendritic cells alone, dendritic cells + GE keratinocytes, and dendritic cells + T-cells cultures, suggesting the presence of both T-cells and GE keratinocytes caused a decrease in the production of MMP9 by dendritic cells in response to HagB. 

Like the single-cell cultures above, there was little to no production of MMP7 and MMP12 in the transwell co-cultures in response to HagB, as there was no significant difference from the no cell control at 64 h (Figure 3b and Figure 5b). However, from the detection of MMP7 and MMP12 from 0 to 32 h, it seems dendritic cell cultures may have been the main producer of MMP12 as it was produced in all cultures containing dendritic cells (Figure 3a and Figure 5a). Dendritic cells + GE keratinocytes and dendritic cells + GE keratinocytes + T-cells produced the greatest amount of MMP7 (Figure 3). MMP7 and MMP12 responses were suggestively enhanced in the dendritic cells + GE keratinocytes + T-cells three-cell transwell co-culture relative to the single-cell cultures, although that enhancement was not significant (Figure 3 and Figure 5).

## 3. Discussion

There has been little research on characterizing MMP responses of cells grown in three-cell transwell co-culture systems using primary GE keratinocytes from the oral cavity. We used the three-cell transwell co-cultures to elucidate cell-to-cell communication that regulates MMP production induced by proinflammatory agonists such as HagB. Co-cultures produced significantly different MMP responses than single-cell cultures, and cytokine and chemokine responses also differed [12].

In this model, dendritic cells drove MMP production, but the expression of MMPs changed in the presence of GE keratinocytes and T-cells. Using transwell co-cultures, we demonstrated how the MMP responses of three cell types differed in a co-culture environment when exposed to the proinflammatory agonist HagB. This experimental approach highlights the contribution and influence multiple cell types in co-culture can have on the MMP response, which plays a role in the pathogenesis of periodontal tissue destruction. Studying the MMP response in this model provides insight into intra- and inter-cellular regulation of MMPs and the role of multiple cell types in the overall MMP inflammatory response. 

Knowing more about the MMP response of the immune system can provide us with a better understanding of regulation of MMP expression. For example, MMP9 cleaves and activates interleukin-8 (IL8), a cytokine that enhances neutrophil recruitment to the site of infection [13]. MMP9 has a major role in extracellular matrix degradation and tissue remodeling [14]. HagB induced a high MMP9 response from dendritic cells in this study. MMP9 is upregulated during wound healing, development, and in inflammatory pathologies. Furthermore, in inflammatory diseases such as arthritis, cardiovascular disease, cancer, and diabetes, MMP9 stimulates the immune response initiating pathogenesis and increasing disease progression. TNFα causes upregulation of MMP9 expression [14]. MMP9 can also recruit eosinophils and T-cells to the site of infection. In this study, the dendritic cell MMP9 response was significantly decreased in the presence of T-cells and GE keratinocytes when all three cell types were cultured together, but not when dendritic cells were cultured with GE keratinocytes or T-cells (Figure 4b). The presence of both GE keratinocytes and T-cells caused an attenuation of the dendritic cell response, suggesting a synergistic inhibition effect of MMP9 expression to prevent further immune cell recruitment. One possibility is that T-cells signal the GE keratinocytes to produce tissue inhibitors of metalloproteinases, which in turn leads to a reduction in MMP9 production, but this has yet to be shown. 

Similarly, dendritic cells produced more collagenase MMP1 than other cell types, but this response was significantly attenuated when all three cell types were cultured together (Figure 2b). MMP1 is elevated during tissue remodeling, wound healing, and repair [15]. Therefore, we expected MMP1 expression to be enhanced by the presence of an agonist like HagB, which occurred in the dendritic cell single-cell culture. When cultured with GE keratinocytes, the dendritic cells’ response is significantly attenuated. This suggests GE keratinocytes may inhibit MMP1 expression in dendritic cells. One possibility is that the GE keratinocytes are inhibiting one of the cytokine inducers of MMP1 expression such as growth factors and interleukins [15].

Co-culture models differ from other systems because of their unique ability to focus on the cellular communication between different cell types over time. Standard *in vitro* culture systems are limited by the inability to represent all of the immune mediators found in the host environment. In particular, a more complex multi-dimensional system that can more closely mimic the *in vivo* inflammatory response is required to generate a comprehensive understanding of the process underlying periodontal disease [16]. There are numerous co-culture models, each with their own intrinsic advantages and disadvantages, which have been extensively described in the current literature [17,18,19,20,21,22,23]. These models generally consist of autologous cells cultured together (e.g., organoids, spheroids, etc.), or allogeneic cells cultured together but physically separated (e.g., transwell co-cultures, etc.). They can be constructed as 2D or 3D systems and may involve different cell types, different types of membranes, and different sources of collagen or matrix. 

The three-cell transwell co-culture system developed for this study is not without its own advantages and disadvantages. We used a heterotypic, transwell co-culture system that does not allow contact among the different cell types by separation using membranes as barriers between the cells. By removing cell–cell physical interactions, we can focus on the effects of cell signals elicited by the agonist or from other cells in the co-culture. Preventing cell-to-cell contact allows us to use cells from different individuals that would otherwise be incompatible. One limitation of this study is that the cells are not necessarily in the same ratios as they would be *in vivo*. With this in mind, we chose to use equal concentrations of each cell type because it is easily repeatable and can be plated in the transwell plates at a reasonable density.

In conclusion, a number of factors could be causing the attenuation of dendritic cell expression of MMP9 and MMP1 in the three-cell co-culture. This study provided insight into MMP regulation in the host environment and the effects of a proinflammatory agonist, HagB. Learning more about these signaling pathways and MMP involvement may lead to identification of possible targets for therapeutic approaches. Further analysis will look for connections between the MMP responses over time and the associated cytokine responses, which will provide more information on the inflammatory response. 

In silico models using bioinformatics are being developed to help us study and analyze inflammatory responses in environments like the periodontal tissues where multiple factors are involved [24]. Previously, we used a computational model to examine the HagB-induced MMP response [10]. Similarly, the results from this study will be used to modify this computational model so that it can be used to search drug databases for potential MMP inhibitors. These drugs can then be tested in the three-cell transwell co-culture to verify their effectiveness in suppressing MMP and inflammatory cytokine expression. This will help us identify and test possible therapeutic approaches for suppressing the inflammatory response in diseases such as periodontal disease.

## 4. Materials and Methods

### 4.1. Preparation of HagB

Hemagglutinin B (HagB) from *P. gingivalis (Pg strain 381)* was cloned and His-tagged into the vector pQE31 and expressed in *Escherichia coli* (M15 pREP4 pQE-31-TX1, QIAGEN Inc., Valencia, CA, USA), which was obtained and prepared similarly to what was previously described [25]. Briefly, recombinant *E. coli* was grown at 37 °C in selective Luria-base (LB) broth with ampicillin and kanamycin A. After 4 h, 1 mM Isopropyl B-D-1-thiogalactopyranoside (IPTG) per liter of culture was added to induce HagB expression. After an additional 4 h of incubation, 10.0 mL of 1.54 M sodium azide (NaN_3_) per liter of culture was added to arrest growth. The culture was pelleted and washed in 10.0 mL of 140 mM phosphate buffered saline (PBS). The PBS was removed, and the cells were resuspended in 10 mL of Buffer A (6.0 M Guanidine-HCl, 0.1 M NaH_2_PO_4_, 0.01 M Tris acid, pH 8.0) to lyse the *E. coli*. The cells were pelleted and the supernatant containing the HagB was collected.

To purify the HagB, affinity chromatography was performed using the BioRad Biologic low-pressure pump and a column containing Profinity IMAC Ni-charged resin (Bio-Rad Laboratories, Inc., Hercules, CA, USA), which binds His-tagged proteins. After the column was equilibrated with Buffer A, the supernatant containing HagB was passed through the column. This elution was collected and discarded since the proteins remained in the column. The affinity-attached HagB was washed with 8.0 M Urea, 0.1 M NaH_2_PO_4_, 0.01 M Tris acid, pH 8.0, followed by a second wash with 8.0 M Urea, 0.1 M NaH_2_PO_4_, 0.01 M Tris acid, pH 6.3. To refold the HagB, a buffer containing 6.0 M Urea, 0.5 M NaCl, 0.01 M Tris acid, 20.0% glycerol, pH 7.4 was applied to the column. The next refolding buffer applied was the same buffer but with 3.0 M Urea followed by buffer without Urea. The last buffer (0.25 M Imidazole, 0.5 M NaCl, 0.01 M Tris acid, 20.0% glycerol, pH 7.4) added to the column was a refolding buffer with imidazole to elute the HagB using a fraction collector. The protein concentration of each fraction was obtained using a NanoDrop (NanoDrop Technologies, Inc., Wilmington, DE, USA), and fractions containing the most protein were pooled.

This protein sample was applied to a desalting column (Bio-Rad Laboratories, Inc., Hercules, CA, USA) to remove imidazole and for further purification using the BioRad Biologic low-pressure pump. The column was equilibrated with a refolding buffer of 0.5 M NaCl, 0.01 M Tris acid, 20.0% glycerol, pH 7.4. The pooled fraction sample was run through the column followed by the refolding buffer without urea. The protein concentration was quantified using a NanoDrop, and fractions containing the most protein were pooled together.

### 4.2. Analysis of HagB

To determine the protein concentration of the sample more precisely, a BCA protein concentration assay was utilized, and found to be approximately 0.445 mg/mL (Pierce bicinchoninic acid (BCA) Protein Assay Kit, ThermoFisher Scientific, Rockford, IL, USA). The final concentration of LPS was 2.44 ng LPS/1.0 µg HagB (QCL-1000, Chromogenic Limulus Amebocyte Lysate Assay, Lonza, Inc. Walkersville, MD, USA). An SDS-Page of the sample was performed to verify purification of HagB using a NuPAGE Novex 4% to 12% Bis-Tris protein gel. This produced a solid band at about 46 kDa. A MALDI-TOF mass spectrometry analysis confirmed the presence of HagB in this band with the greatest number of matches and sequences of all proteins found. HagB was the only protein found from *P. gingivalis*. 

### 4.3. Dendritic cells, GE keratinocytes, and T-cells

Dendritic cells, GE keratinocytes, and T-cells were chosen for this experiment due to their presence in the periodontium and their innate immune functions. Human monocyte-derived immature myeloid dendritic cells (AllCells, Alameda, CA, USA) were thawed, washed with LGM-3 (Lymphocyte Growth Media-3, Lonza, Walkersville, MD, USA), counted, pelleted by centrifugation (400× *g*) at 4 °C for 10 min, and then resuspended in LGM-3 to 1 × 10^5^ viable cells/mL. CD4+ T-cells (StemCell Technologies, Inc., Vancouver, BC, Canada) were activated using a human T-cell Activation/Expansion kit (Miltenyi Biotec, San Diego, CA, USA) in TexMACS medium (Miltenyi Biotec) for 3 days at 37 °C with 5% CO_2_. The T-cells were then collected, pooled, and a viable cell count was obtained. The T-cells were resuspended to 1 × 10^5^ viable activated cells/mL in LGM-3. Primary first passage GE keratinocytes were used, which were previously prepared and stored in liquid nitrogen [26]. These cells were isolated from healthy, non-smoking individuals undergoing crown lengthening or canine exposure procedures. In compliance with a protocol approved by the University of Iowa Institutional Review Board for the Use of Human Subjects in Research (199811030, approval date 11 June 2005), informed consent was previously obtained from these patients.

All three cell types were centrifuged at 400× *g* at 4 °C for 10 min and resuspended in LGM-3 to 1 × 10^5^ viable cells/mL. LGM-3 was used because all three cell types can survive and remain active in this serum-free media.

### 4.4. Establishment of Co-Culture

The three-cell transwell co-culture system was made using transwell 12-well polystyrene plates (3104, Corning, Inc., Corning, NY, USA) and inserts from the transwell 24-well polystyrene plates (3413, Corning, Inc., Corning, NY, USA). 200 µL of 1 × 10^5^ cells/mL of dendritic cells were added to the bottom well of the transwell 12-well plate along with 800 µL of LGM-3. 200 µL of 1 × 10^5^ cells/mL of GE keratinocytes were added to the middle 12 mm insert along with 300 µL of LGM-3. 200 µL of 1 × 10^5^ cells/mL of activated T-cells were added to the top 6.5 mm insert. These inserts have a permeable membrane with a pore size of 0.4 um to allow media to be shared between the three cell types. The cell layers are organized in this manner due to cell properties. For example, dendritic cells are placed in the bottom well so they can spread out and adhere to the plate. GE Keratinocytes also somewhat adhere so they were given the middle tier, while the T-cells do not adhere and therefore do not require as much surface area on the plate. The plates were placed on a plate shaker at low speed in the incubator at 37 °C with 5% CO_2_ (Figure 1a). This created a diffusible 3-tier platform. The total volume was 1700 µL/well.

To compare the responses between different cell types, single-cell responses, and the response of all three cell types together, combinations of cell types and single cells were set up in the same manner (Figure 1b). In total, there were three replicates of each well. LGM-3 was added in place of cell suspensions not present in a well. The HagB diluent (refolding buffer: 0 M Urea, 0.5 M NaCl, 0.01 M Tris acid, 20% glycerol, pH 7.4) was used as the treatment control, which allows any responses from the buffer to be subtracted out. The plates were incubated for 2 h at 37 °C to allow the cells to settle and adjust.

HagB was then added at a concentration of 10.0 µg/mL. Preliminary tests were used to determine the appropriate concentration of HagB to use by testing varying concentrations and their respective dendritic cell response. An equal amount of buffer was added to control wells. Plates were placed on a titer plate shaker at a low speed for 60 s to diffuse the treatment. 200 µL of supernatant was collected from each well as a time zero sample, and 200 µL of LGM-3 was added back to each well. Samples were centrifuged for five minutes at 0.8× *g* to pellet any cells or debris, and supernatants were stored at −80 °C. The plates were placed on the shaker and incubated at 37 °C, 5% CO_2_ for 64 h. Samples were collected in this same manner at 2, 4, 8, 16, and 32 h. At 64 h, 2.0 mL centrifugal filter tubes were used to pool the media, one tier at a time. 

### 4.5. Determination of MMP Concentrations

The concentrations of MMP1, MMP7, MMP9, and MMP12 were first evaluated to examine production over 32 h. This was done using multiplex immunoassays (Human Magnetic Luminex Screening Assay, R & D Systems, Minneapolis, MN, USA) read on the Luminex^100^ (Luminex 100 IS Instrument, Luminex, Austin, TX, USA) (*n* = 3) (Figure 2a, Figure 3a, Figure 4a and Figure 5a). After the experiment was repeated twice more, MMP concentrations at 64 h were further evaluated and compared (*n* = 9) using multiplex immunoassays (Milliplex MAP Human MMP Magnetic Bead Panels, EMD Millipore, Billerica, MA, USA) read on the Luminex^100^ (Figure 2b, Figure 3b, Figure 4b and Figure 5b). These immunoassay kits use antibody-coated magnetic beads to bind desired analytes and use a standard curve of known concentrations to determine unknown concentrations as previously described [27].

### 4.6. Statistical Analysis

MMP concentrations were interpolated from curves constructed from the MMP standards and their respective median fluorescence intensity (MFI) readings on the Luminex^100^ and readout files (Milliplex Analyst v5.1 software, EMD Millipore, Billerica, MA, USA). First, the 0 to 32 h results from the immunoassay (Human Magnetic Luminex Screening Assay, R & D Systems, Minneapolis, MN, USA) were analyzed (*n* = 3). The responses of the control plate (HagB diluent) were subtracted from the responses of the test plate (HagB) to assess only the effect of the presence of HagB. A two-way fixed effect ANOVA was fit to log-transformed concentrations of the MMPs (Analysis performed using JMP, Version 10.0, SAS, Cary, NC, USA). Pairwise group comparisons were conducted using the post-hoc Tukey’s honest significance test. A significance level of 0.05 was used determine statistically significant differences between groups.

The co-culture experiment was then repeated three times, and three repeats from each experiment at 64 h were included in the immunoassay (*n* = 9). Responses from the control plate (HagB diluent) were subtracted from the responses from the test plate (HagB) to assess only the effect of HagB. Once again, results were analyzed using a two-way fixed effect ANOVA fit to log-transformed data and pairwise group comparisons were made.

## Supplementary Materials

Supplementary materials can be found at http://www.mdpi.com/1422-0067/19/12/3923/s1.
