# Supplementary material for: Matrix Metalloproteinase Response of Dendritic Cell, Gingival Epithelial Keratinocyte, and T-Cell Transwell Co-Cultures Treated with Porphyromonas gingivalis Hemagglutinin-B
Source: Int J Mol Sci. 2018 Dec 7;19(12):3923. doi: 10.3390/ijms19123923 (PMC6321455; doi:10.3390/ijms19123923)
Supplement: Supplementary file 1 [file ijms-19-03923-s001.pdf]

Supplemental Table S1. HagB-induced MMP log10 responses at all time points (N=3). Means and SEM are shown.

Time (hr)	MMP	No cells	DC	Ker	TC	DC+Ker	DC+TC	Ker+TC	DC+Ker+TC
**0**	**1**	0.2803<br>0.3243<br>A	0.2803<br>0.3243<br>A	0.7380<br>0.3243<br>A	0.7400<br>0.3243<br>A	0.9770<br>0.3243<br>A	0.3680<br>0.3243<br>A	0.3993<br>0.3243<br>A	0.3310<br>0.3243<br>A
**2**	**1**	0.4247<br>0.2581<br>A	0.0000<br>0.2581<br>A	0.0000<br>0.2581<br>A	0.0000<br>0.2581<br>A	0.5937<br>0.2581<br>A	0.0000<br>0.2581<br>A	0.0000<br>0.2581<br>A	0.0000<br>0.2581<br>A
**4**	**1**	0.2647<br>0.2559<br>A	0.0000<br>0.2559<br>A	0.2693<br>0.2559<br>A	0.8990<br>0.2559<br>A	0.8303<br>0.2559<br>A	0.7203<br>0.2559<br>A	0.9993<br>0.2559<br>A	0.9977<br>0.2559<br>A
**8**	**1**	0.3100<br>0.2981<br>AB	1.1507<br>0.2981<br>AB	0.6023<br>0.2981<br>AB	0.0000<br>0.2981<br>B	1.2947<br>0.2981<br>AB	1.4060<br>0.2981<br>AB	1.2097<br>0.2981<br>AB	1.5147<br>0.2981<br>A
**16**	**1**	0.2953<br>0.1944<br>B	2.3007<br>0.1944<br>A	0.0000<br>0.1944<br>B	0.0000<br>0.1944<br>B	2.1277<br>0.1944<br>A	2.0523<br>0.1944<br>A	0.0000<br>0.1944<br>B	2.2083<br>0.1944<br>A
**32**	**1**	0.0000<br>0.4244<br>B	2.4210<br>0.4244<br>A	0.0000<br>0.4244<br>B	0.7493<br>0.4244<br>AB	1.6320<br>0.4244<br>AB	2.3457<br>0.4244<br>A	0.0000<br>0.4244<br>B	1.3073<br>0.4244<br>AB
**64**	**1**	0.5613<br>0.4254<br>AB	2.2003<br>0.4254<br>A	0.0000<br>0.4254<br>B	0.2553<br>0.4254<br>AB	1.4200<br>0.4254<br>AB	1.5467<br>0.4254<br>AB	0.0000<br>0.4254<br>B	0.3667<br>0.4254<br>AB
**0**	**7**	0.1540<br>0.3399<br>A	0.2997<br>0.3399<br>A	1.2627<br>0.3399<br>A	0.8850<br>0.3399<br>A	0.7553<br>0.3399<br>A	0.3967<br>0.3399<br>A	0.3777<br>0.3399<br>A	0.7553<br>0.3399<br>A
**2**	**7**	0.0000<br>0.1455<br>A	0.0000<br>0.1455<br>A	0.0000<br>0.1455<br>A	0.0000<br>0.1455<br>A	0.4117<br>0.1455<br>A	0.0000<br>0.1455<br>A	0.0000<br>0.1455<br>A	0.0000<br>0.1455<br>A
**4**	**7**	0.4363<br>0.3769<br>A	0.4360<br>0.3769<br>A	0.0000<br>0.3769<br>A	1.3087<br>0.3769<br>A	0.0000<br>0.3769<br>A	0.5333<br>0.3769<br>A	0.8723<br>0.3769<br>A	1.0270<br>0.3769<br>A
**8**	**7**	0.0000<br>0.3165<br>A	0.3210<br>0.3165<br>A	0.6497<br>0.3165<br>A	0.3210<br>0.3165<br>A	0.6793<br>0.3165<br>A	0.3210<br>0.3165<br>A	1.4180<br>0.3165<br>A	0.4533<br>0.3165<br>A
**16**	**7**	0.1517<br>0.3601<br>A	0.4670<br>0.3601<br>A	0.7413<br>0.3601<br>A	0.3840<br>0.3601<br>A	1.3543<br>0.3601<br>A	1.0653<br>0.3601<br>A	0.3037<br>0.3601<br>A	1.0033<br>0.3601<br>A
**32**	**7**	0.4083<br>0.4235<br>A	1.5097<br>0.4235<br>A	0.7137<br>0.4235<br>A	0.6860<br>0.4235<br>A	1.7680<br>0.4235<br>A	1.4417<br>0.4235<br>A	0.9217<br>0.4235<br>A	2.0017<br>0.4235<br>A
**64**	**7**	0.0000<br>0.4528<br>B	0.4303<br>0.4528<br>AB	0.6190<br>0.4528<br>AB	0.0000<br>0.4528<br>B	2.2620<br>0.4528<br>A	0.6687<br>0.4528<br>AB	0.7683<br>0.4528<br>AB	2.2633<br>0.4528<br>A
**0**	**9**	0.2047<br>0.3684<br>A	0.9583<br>0.3684<br>A	0.1033<br>0.3684<br>A	0.2637<br>0.3684<br>A	1.1497<br>0.3684<br>A	0.2993<br>0.3684<br>A	0.3757<br>0.3684<br>A	0.3543<br>0.3684<br>A
**2**	**9**	0.1530<br>0.3094<br>A	0.0000<br>0.3094<br>A	0.0000<br>0.3094<br>A	0.0000<br>0.3094<br>A	0.8033<br>0.3094<br>A	0.0000<br>0.3094<br>A	0.0000<br>0.3094<br>A	0.3113<br>0.3094<br>A
**4**	**9**	0.6103<br>0.2543<br>BCD	1.5637<br>0.2543<br>ABC	0.1793<br>0.2543<br>D	0.2450<br>0.2543<br>D	1.8540<br>0.2543<br>AB	1.9553<br>0.2543<br>A	0.5677<br>0.2543<br>CD	1.8610<br>0.2543<br>A
**8**	**9**	0.0000<br>0.5506<br>A	1.5753<br>0.5506<br>A	0.0000<br>0.5506<br>A	0.0000<br>0.5506<br>A	1.7470<br>0.5506<br>A	1.7813<br>0.5506<br>A	0.4510<br>0.5506<br>A	2.5003<br>0.5506<br>A

**16**	**9**	0.0000<br>0.0704<br>B	3.2963<br>0.0704<br>A	0.0000<br>0.0704<br>B	0.0000<br>0.0704<br>B	3.2743<br>0.0704<br>A	3.2163<br>0.0704<br>A	0.0000<br>0.0704<br>B	3.2897<br>0.0704<br>A
**32**	**9**	0.0000<br>0.2082<br>B	3.6690<br>0.2082<br>A	0.3777<br>0.2082<br>B	0.1817<br>0.2082<br>B	3.8343<br>0.2082<br>A	3.6657<br>0.2082<br>A	0.0000<br>0.2082<br>B	3.4720<br>0.2082<br>A
**64**	**9**	0.0000<br>0.1901<br>B	4.2337<br>0.1901<br>A	0.0000<br>0.1901<br>B	0.2150<br>0.1901<br>B	4.3033<br>0.1901<br>A	4.0490<br>0.1901<br>A	0.4570<br>0.1901<br>B	4.1267<br>0.1901<br>A
**0**	**12**	0.0890<br>0.1464<br>A	0.0000<br>0.1464<br>A	0.1613<br>0.1464<br>A	0.1723<br>0.1464<br>A	0.0000<br>0.1464<br>A	0.1613<br>0.1464<br>A	0.0110<br>0.1464<br>A	0.2950<br>0.1464<br>A
**2**	**12**	0.0000<br>0.3118<br>A	0.7890<br>0.3118<br>A	0.0000<br>0.3118<br>A	0.0000<br>0.3118<br>A	0.6590<br>0.3118<br>A	0.9170<br>0.3118<br>A	0.0000<br>0.3118<br>A	0.8067<br>0.3118<br>A
**4**	**12**	0.1500<br>0.3039<br>A	1.0527<br>0.3039<br>A	0.0953<br>0.3039<br>A	0.0000<br>0.3039<br>A	1.0403<br>0.3039<br>A	1.0770<br>0.3039<br>A	0.0000<br>0.3039<br>A	0.9947<br>0.3039<br>A
**8**	**12**	0.3727<br>0.3687<br>A	1.2383<br>0.3687<br>A	0.1060<br>0.3687<br>A	0.0000<br>0.3687<br>A	1.5007<br>0.3687<br>A	1.4783<br>0.3687<br>A	0.4480<br>0.3687<br>A	1.5070<br>0.3687<br>A
**16**	**12**	0.0013<br>0.4296<br>A	1.5533<br>0.4296<br>A	0.3033<br>0.4296<br>A	0.0000<br>0.4296<br>A	1.7173<br>0.4296<br>A	1.3167<br>0.4296<br>A	0.1767<br>0.4296<br>A	1.4183<br>0.4296<br>A
**32**	**12**	0.1763<br>0.2988<br>D	1.6053<br>0.2988<br>ABCD	0.2493<br>0.2988<br>CD	0.1763<br>0.2988<br>D	1.7157<br>0.2988<br>AB	1.6447<br>0.2988<br>ABC	0.2937<br>0.2988<br>BCD	1.7613<br>0.2988<br>A
**64**	**12**	0.0000<br>0.3549<br>B	1.2747<br>0.3549<br>AB	0.2910<br>0.3549<br>AB	0.0000<br>0.3549<br>B	1.6913<br>0.3549<br>AB	1.3647<br>0.3549<br>AB	0.2597<br>0.3549<br>AB	1.8563<br>0.3549<br>A

Supplemental Table S1. HagB-induced MMP log10 responses at 0, 2, 4, 8, 16, 32, and 64 hours (N=3). Means and SEM are shown. The responses of the control plate (HagB diluent) were subtracted from the responses of the test plate (HagB) to assess only the effect of the presence of HagB in single cell cultures and co-cultures of T-cells (TC), GE keratinocytes (Ker), and dendritic cells (DC). A two-way fixed effect ANOVA was fit to log-transformed concentrations of the MMPs. Pairwise group comparisons were conducted using the post-hoc Tukey's Honest Significant Differences test. A 0.05 level was used determine statistically significant differences between groups. Values with the same letter are not significantly different. Differing letters indicate significantly different means ($p<0.05$).

Supplemental Table S2. 64 hour HagB-induced MMP log10 responses (N=9). Means (SEM) are shown.								
**MMP**	**No cells**	**DC**	**Ker**	**TC**	**DC+Ker**	**DC+TC**	**Ker+TC**	**DC+Ker+TC**
**MMP1**	0.123<br>(0.219)<br>B	1.647<br>(0.219)<br>A	0<br>(0.219)<br>B	0.001<br>(0.219)<br>B	0.415<br>(0.219)<br>B	0.895<br>(0.219)<br>A,B	0<br>(0.219)<br>B	0<br>(0.219)<br>B
**MMP7**	0.569<br>(0.429)<br>A,B	1.067<br>(0.429)<br>A,B	1.056<br>(0.429)<br>A,B	0<br>(0.429)<br>B	1.937<br>(0.429)<br>A	0.646<br>(0.429)<br>A,B	1.106<br>(0.429)<br>A,B	1.885<br>(0.429)<br>A,B
**MMP9**	0.219<br>(0.233)<br>C	3.385<br>(0.233)<br>A	0<br>(0.233)<br>C	0.342<br>(0.233)<br>C	3.315<br>(0.233)<br>A	3.114<br>(0.233)<br>A	0.337<br>(0.233)<br>C	1.704<br>(0.233)<br>B
**MMP12**	0.346<br>(0.272)<br>A,B	0.960<br>(0.272)<br>A,B	0.375<br>(0.272)<br>A,B	0.008<br>(0.272)<br>B	1.284<br>(0.272)<br>A	1.162<br>(0.272)<br>A,B	0.709<br>(0.272)<br>A,B	1.405<br>(0.272)<br>A

Supplemental Table S2. 64 hour HagB-induced MMP log10 responses (N=9). Means and SEM are shown. The responses of the control plate (HagB diluent) were subtracted from the responses of the test plate (HagB) to assess only the effect of the presence of HagB in single cell cultures and co-cultures of T-cells (TC), GE keratinocytes (Ker), and dendritic cells (DC). A two-way fixed effect ANOVA was fit to log-transformed concentrations of the MMPs. Pairwise group comparisons were conducted using the post-hoc Tukey's Honest Significant Differences test. A 0.05 level was used determine statistically significant differences between groups. Values with the same letter are not significantly different. Differing letters indicate significantly different means ($p<0.05$).
